# Changes in Cortical Activity during Preferred and Fast Speed Walking under Single- and Dual-Tasks in the Young-Old and Old-Old Elderly

**DOI:** 10.3390/brainsci11121551

**Published:** 2021-11-23

**Authors:** Jinuk Kim, Gihyoun Lee, Jungsoo Lee, Yun-Hee Kim

**Affiliations:** 1 Department of Physical and Rehabilitation Medicine, Center for Prevention and Rehabilitation, Heart Vascular Stroke Institute, Samsung Medical Center, Sungkyunkwan University School of Medicine, Seoul 06351, Korea; kimjuk92@gmail.com (J.K.); gihyounlee@gmail.com (G.L.); 2 Department of Health Sciences and Technology, SAIHST, Sungkyunkwan University, Seoul 06355, Korea; 3 Department of Medical Device Management & Research, Department of Digital Health, SAIHST, Sungkyunkwan University, Seoul 06355, Korea

**Keywords:** functional near-infrared spectroscopy, cortical activity, gait, dual-task walking, aging

## Abstract

In the elderly, walking while simultaneously engaging in other activities becomes more difficult. This study aimed to examine the changes in cortical activity during walking with aging. We try to reveal the effects of an additional task and increased walking speed on cortical activation in the young-old and the old-old elderly. Twenty-seven young-old (70.2 ± 3.0 years) and 23 old-old (78.0 ± 2.3 years) participated in this study. Each subject completed four walking tasks on the treadmill, a 2 × 2 design; two single-task (ST) walking conditions with self-selected walking speed (SSWS) and fast walking speed (FWS), and two dual-task (DT) walking conditions with SSWS and FWS. Functional near-infrared spectroscopy was applied for measurement of cerebral oxyhemoglobin (oxyHb) concentration during walking. Cortical activities were increased during DT conditions compared with ST conditions but decreased during the FWS compared with the SSWS on the primary leg motor cortex, supplementary motor area, and dorsolateral prefrontal cortex in both the young-old and the old-old. These oxyHb concentration changes were significantly less prominent in the old-old than in the young-old. This study demonstrated that changes in cortical activity during dual-task walking are lower in the old-old than in the young-old, reflecting the reduced adaptive plasticity with severe aging.

## 1. Introduction

In the elderly, walking performance starts to decline [1,2]. The representative gait pattern characteristic of aging is a decrease in gait speed, step length, and swing phase and an increase in step width, double support time, and gait variability [2]. It has been reported that the decrease in walking ability with aging increases the risk of falls, decreases the activities of daily living and quality of life, and has a high association with mortality [1,3,4].

There is growing interest in the influence of age-related decline in gait capacity on brain function [5]. Gait is influenced by higher-order cognitive and cortical control mechanisms [6]. The central motor networks, including primary motor (M1), premotor (PM), and prefrontal (PFC) cortices, are activated during walking [6]. Gait characteristics such as speed and variability are related closely to the cortical activity regulated by multiple supraspinal control mechanisms [7]. Slow gait speed in the elderly is related to the frontoparietal control network, which is related to executive function, and gait stability is related to the function of the dorsal attention network [8,9]. However, these studies did not measure real-time functional brain activity during actual walking tasks but rather confirmed the neural correlates of gait using conventional neuroimaging tools such as functional magnetic resonance imaging (fMRI) and positron emission tomography (PET). Functional near-infrared spectroscopy (fNIRS) has an advantage of being useful for measuring walking or activities of daily living because it is a relatively small, portable structure. Unlike fMRI and PET, fNIRS is silent and has no safety concerns [10,11]. Recently, many studies have been conducted to measure real-time brain activation during walking using fNIRS [12].

Walking while simultaneously engaging in other activities becomes more and more difficult with increasing age and causes frequent falls [13]. A dual-task walking paradigm, which performs secondary tasks such as talking or counting while walking, offers the advantage of directly assessing the causal impact of attention resources on gait [14]. Previous studies have reported that dual-task costs (i.e., the difference in performance between single- and dual-task conditions) are greater in older adults than in younger adults, and a greater decrease in walking speed in older adults than in younger adults is frequent [15,16]. Additional secondary tasks can interfere with central motor network activation as well as gait ability, especially in older adults [14,17]. In previous fNIRS studies, increased PFC activation during dual-task walking was described in various participant groups, including young adults and the elderly [10,18]. However, although the dual-task cost was higher in the elderly than in young adults, the difference in cortical activity is not clear [18,19]. Also, most studies have been limited to only PFC activity during dual-task walking, and activity in other cortices involved in the central motor network, such as the PM, supplementary motor area (SMA), somatosensory motor cortex (SMC), and posterior parietal cortex (PPC), has yet to be reported. Therefore, it is necessary to confirm the effect of age in areas other than the PFC even during dual-task walking in the elderly.

The decline in mobility is accelerated with age in the elderly [20,21] and this acceleration is evident over 75 years of age. It has been reported that the old-old elderly over 75 walk much slower with shorter steps and increased step width compared to the young-old [22]. Therefore, geriatric studies often investigated the characteristics of ageing by dividing the elderly into two distinct groups; the young-old (65–74 years old) and the old-old (75 years old and older) groups [23]. Walking ability and dual-task performance are also lower in the old-old than in the young-old [24,25]. Therefore, it can be helpful to understand the characteristics of aging by dividing the young-old elderly and the old-old elderly for examining the intergroup differences.

This study aimed to examine the changes in cortical activity during walking with aging. Using multi-channel fNIRS measurement equipment, a wider cortex including not only the frontal lobe, but also the motor area and parietal lobe was measured during walking. Through this, we analyzed the effects of a simultaneous task and increased walking speed on cortical activation in the young-old and old-old groups. We hypothesized that cortical activity would change not only in the PFC but also in other cortical areas during dual-task or fast speed walking compared to single-task walking at self-selected walking speed in the elderly. We also hypothesized that these changes would appear differently between the young-old and the old-old groups.

## 2. Materials and Methods

### 2.1. Participants

Fifty elderly participants aged 65 to 84 years (19 males; mean age: 73.8 ± 4.8 years; range: 65–82 years) without a history of neurological or psychiatric symptoms participated in this cross-sectional study. Based on age, they were divided into two groups: the young-old elderly from 65 to 74 years (*n* = 27, 10 males; mean age: 70.2 ± 3.0 years) and the old-old elderly from 75 to 84 years (*n* = 23, nine males; mean age: 78.0 ± 2.3 years). Participants were excluded if they had (1) difficulty walking independently due to problems such as visual field loss, (2) a history of musculoskeletal disorders such as fracture, muscle or nerve injuries that can cause problems with lower extremity functions within 3 months prior to enrollment, or (3) severe cognitive decline with a score of 10 or less on the Korean Mini-Mental State Exam (K-MMSE) [26]. This experimental protocol was approved by the Institutional Review Board of Samsung Medical Center (2020-09-172), and all participants provided informed consent.

### 2.2. Study Design and Experimental Protocol

All participants who agreed to participate in the study were investigated for sociodemographic characteristics (age, sex, and level of education), height, weight, body mass index (BMI), and medical history. Evaluations such as the short physical performance battery (SPPB) [27], the four-square step test (FSST) [28], the timed up and go test (TUG) [29], and the 10-m walking test (10MWT) [30] were performed to assess the participant’s physical and gait functions. The K-MMSE to evaluate cognitive function [31], geriatric depression scale short form (GDS-SF) to measure depression [32], and EuroQol-5 dimension (EQ-5D) and Korean-modified Barthel index (K-MBI), which evaluate the levels of activities of daily living, also were performed [33,34].

Each participant completed four walking conditions on the treadmill, a 2×2 within-subjects factorial design (task × speed): single-task walking conditions (ST conditions) with self-selected walking speed (SSWS) and fast walking speed (FWS) and dual-task walking conditions (DT conditions) with SSWS and FWS. The walking trials were arranged in a block paradigm. For each trial, walking tasks consisted of a baseline in a standing state for 30 s before the start and five task blocks (30 s in duration) alternating with five rest blocks (30 s in duration) for a total of 330 s. To avoid fatigue between conditions, subjects were given sufficient rest for 5 to 10 min before each condition was started. The SSWS was set at the speed at which each subject felt comfortable walking on the treadmill, similar to natural walking of each individual [35]. FWS was set 130% faster than SSWS [36]. Participants were given some time to adapt to both speeds, and the measurement began after taking enough rest. During DT conditions, the participants were asked to maintain walking while performing a phonemic verbal fluency task that required saying as many words as possible that began with alternate letters as presented aurally and visually (common consonants) [37]. They were not specifically instructed to prioritize which tasks during DT conditions. To maintain a consistent level of cognitive effort during the task periods, two letters were provided for every 30 s of walking task block. A total of 10 letters were randomly presented during all five blocks, and the total number of responses (words) spoken by the participants were recorded.

### 2.3. Measurement of Functional Near-Infrared Spectroscopy

**Figure 1:**
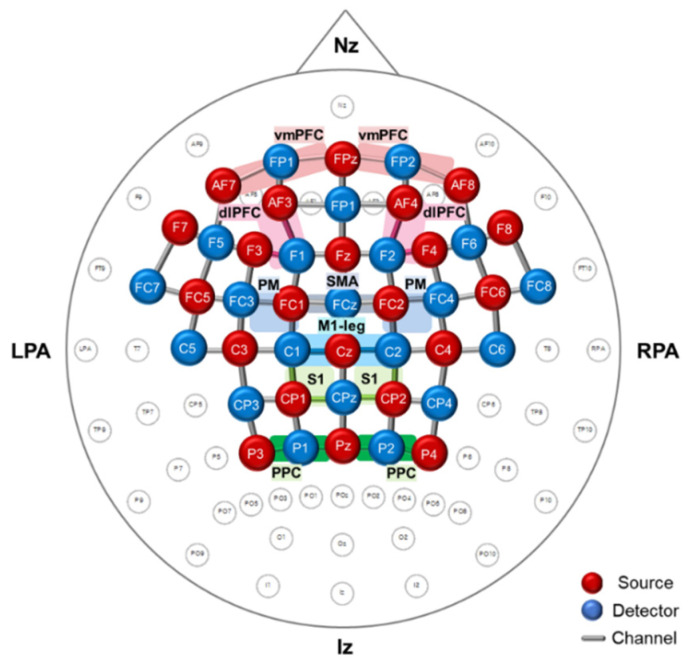
Position of functional near-infrared spectroscopy (fNIRS) measurement optodes and channels. In the fNIRS channel arrangement, 22 sources and 21 detectors covered the cortical areas of the bilateral M1-leg, PM, SMA, S1, PPC, dlPFC, and vmPFC using 71 channels of interest. LPA, left pre-auricular points; RPA, right pre-auricular points; Nz, nasion; Iz, inion; M1-leg, primary leg motor cortex; PM, premotor area; SMA, supplementary motor area; S1, primary somatosensory cortex; PPC, posterior parietal cortex; dlPFC, dorsolateral prefrontal cortex; vmPFC, ventromedial prefrontal cortex.

Cortical activity during treadmill walking was confirmed through oxyhemoglobin (oxyHb) concentration measured by functional near-infrared spectroscopy (fNIRS) system (NIRScout^®^; NIRx Medical Technology, Berlin, Germany). The fNIRS optodes consisted of 22 LED light sources and 21 detectors, and 71 source-detector channels were used for monitoring the hemodynamics (Figure 1). The regions of interest (ROIs) were the bilateral primary leg motor cortex (M1-leg, Brodmann areas (BA 4), bilateral PM (BA 6), SMA (BA 6), bilateral somatosensory motor cortex (S1, BA 3), bilateral PPC (BA 7), bilateral dorsolateral PFC (dlPFC, BA 9), and bilateral ventromedial PFC (vmPFC, BA 11). The fNIRS optodes were positioned according to the international 10/20 system, and the channel distance (i.e., distance between the source and detector) was 3.0 cm. The cranial vertex (Cz) located beneath the first source was the marker for ensuring replicable placement of the optodes. After the Cz position was determined on the participant’s head, an fNIRS head cap was placed. The fNIRS system used two wavelengths, 760 nm and 850 nm, with a sampling rate of 10.42 Hz.

### 2.4. Data Processing and Analysis of Functional Near-Infrared Spectroscopy

fNIRS data were processed using the open-source software NIRS-SPM implemented in MATLAB^®^ (MathWorks Inc., Natick, MA, USA). In statistical parametric mapping (SPM) analysis, a generalized linear model (GLM) with standard hemodynamic response curves was performed to model the hypothesized oxyHb response and examined for significant cortical activation during the experiment [38]. At the group level, statistical analysis was performed based on the individual-level beta-values to identify activated channels (corrected *p* < 0.001) [39]. Furthermore, t-statistic maps computed for group analysis were plotted onto a conventional brain template, and the regions with the significant difference in oxyHb concentration were identified.

Changes in oxyHb concentration were analyzed using the nirsLAB^®^ software (v.2019.04; NIRx Medical Technology, Berlin, Germany). Spike artifacts and discontinuous data in the measurement signal were removed and replaced by the nearest signals. The raw data were first band-pass filtered from 0.01 Hz to 0.2 Hz to remove baseline noise and to eliminate possible respiration and heart rate signals [40]. Both oxyHb and deoxyhemoglobin (deoxyHb) signals were obtained in the measurement, but only oxyHb concentrations were used for analysis due to the superior signal-to-noise ratio. [41]. The oxyHb concentration was calculated from preprocessed filtered data using a modified Beer-Lambert law for each of the 71 channels [42]. For each channel, the grand average of each hemodynamic response was computed. Block averages of the walking trial periods for a 30-s interval without cutting out were performed to extract the integral values of the oxyHb concentration changes. The representative integral values of ROI were obtained from average values of the channels included in each ROI. The coordinates and the target ROIs were decided using the fNIRS Optodes Location Decider (fOLD) toolbox in MATLAB^®^ [43].

### 2.5. Statistical Analysis

Statistical analysis was performed using IBM SPSS 20.0 (IBM Corp., Armonk, NY, USA). Data normality and homogeneity of all variances were confirmed with the Shapiro-Wilk test and Levene’s test, respectively. At first, demographic characterization, functional differences, treadmill speed, and verbal fluency responses between the young-old and the old-old were examined by the independent-samples t-test and Chi-square test. For fNIRS data statistical analysis, the integral values of oxyHb in each ROI were analyzed by conducting a 2 × 2 × 2 mixed-design ANOVA with task (two levels; ST and DT conditions) and speed (two levels; SSWS and FWS) as the independent within-subjects variables and group (two levels; young-old and old-old) as the between-subjects factor. When the task effect, speed effect, or group interaction effect was significant, Bonferroni adjusted post hoc tests were used to analyze pairwise comparisons (i.e., ST versus DT, SSWS versus FWS, and young-old versus old-old). The association of integral values of oxyHb in each ROI and age was explored using Pearson’s correlation coefficients. For all analyses, the level of significance was set at *p* = 0.05.

## 3. Results

### 3.1. Characteristics of the Study Population and Functional Differences between the Young-Old and the Old-Old

Table 1 shows the characteristics of the participants and the differences between the young-old and the old-old. The two groups had statistical differences only in age, and there were no differences in sociodemographic characteristics. As a result of the functional differences between the two groups, the old-old had significantly lower physical and gait functions than the young-old in the SPPB, FSST, TUG, and 10MWT (*p* < 0.05) (Table 1).

Furthermore, the old-old showed significantly lower scores compared to the young-old in GDS-SF and EQ-5D. There were no differences in K-MBI and K-MMSE. On the treadmill, the old-old walked significantly slower than the young-old in both SSWS and FWS (*p* < 0.05) (Table 2). In the DT-conditions, the number of responses in the verbal fluency task was significantly lower in the old-old than the young-old at both speeds (*p* < 0.05) (Table 2).

### 3.2. Cortical Activation Patterns during Walking in the Young-Old and the Old-Old

**Figure 2:**
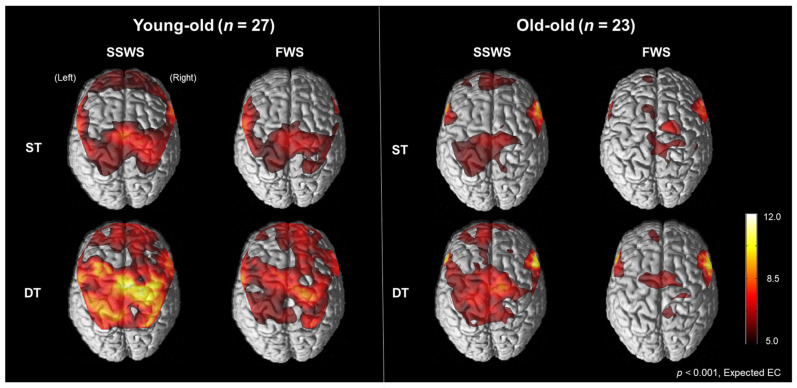
Cortical activation patterns during walking in the young-old and the old-old. Cortical activities measured by oxyHb concentration were increased during dual-task walking compared with single-task walking but decreased during fast-walking speed compared with self-selected walking speed in the bilateral M1-leg, SMA, and dlPFC in both the young-old and the old-old elderly. ST, single-task; DT, dual-task; SSWS, self-selected walking speed; FWS, fast walking speed.

Figure 2 illustrates cortical activation patterns during walking in each condition in the young-old and the old-old. In the SPM analysis results, cortical activation measured by oxyHb concentration was increased during the DT conditions compared with the ST conditions but decreased during the FWS conditions compared with the SSWS conditions on the bilateral M1-leg, SMC, PM, SMA, and dlPFC in both the young-old and the old-old. Cortical activation was lower in the old-old than the young-old.

### 3.3. Regional oxyHb Values during Walking in the Young-Old and the Old-Old

**Figure 3:**
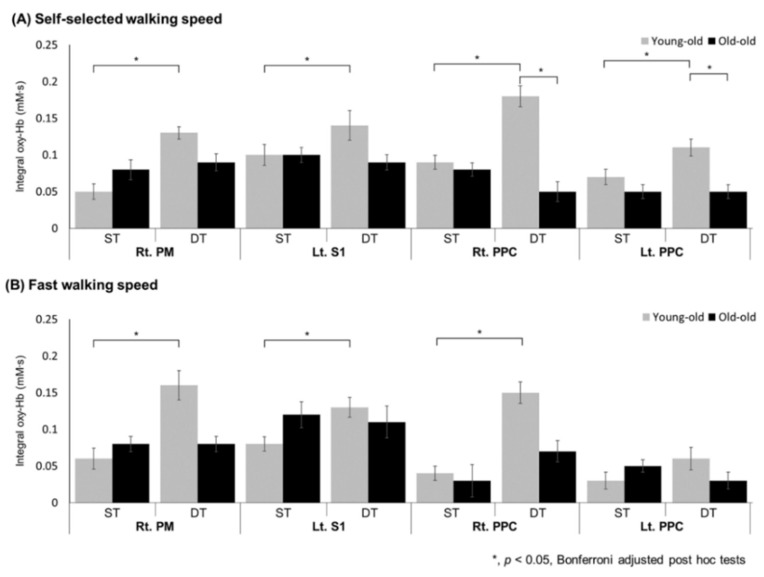
Changes in integral values of oxyHb concentration during walking in the young-old and the old-old. In the young-old, oxyHb significantly increased in the right PM, left S1, and bilateral PPC at both self-selected walking speed (**A**) and fast walking speed (except left PPC) (**B**) during the dual-task walking compared with the single-task. However, there was no change in the old-old elderly. In addition, oxyHb concentration in the bilateral PPC was significantly lower in the old-old elderly compared with the young-old during the dual-task walking. oxyHb, oxyhemoglobin; PM, pre-motor area; S1, primary somatosensory motor cortex; PPC, posterior parietal cortex; ST, single-task; DT, dual-task; Rt., right; Lt., left.

We examined the effects of task, speed, and group on changes in oxyHb concentration in each ROI. The changes in the integral value of oxyHb for each ROI under the two conditions (task and speed) and groups are described in Table 3, and the main effect was confirmed by mixed ANOVA. The main effect of task was found in the right M1-leg, bilateral PM, SMA, right PPC, bilateral dlPFC, and bilateral vmPFC, and there was a significant increase in oxyHb concentration during DT conditions compared with ST conditions (*p* < 0.05). The main effect of speed was found in the left PPC and left vmPFC, and the cortical activity was decreased in FWS conditions compared with SSWS conditions. The interaction main effect between task and group (task × group) was shown in the right PM (F(1, 47) = 9.069, *p* = 0.004, *η*^2^ = 0.162), left S1 (F(1, 47) = 5.808, *p* = 0.020, *η*^2^ = 0.110), and bilateral PPC (right PPC; F(1, 47) = 7.651, *p* = 0.008, *η*^2^ = 0.140, left PPC; F(1, 47) = 4.226, *p* = 0.045, *η*^2^ = 0.083). There was no interaction between speed and group (speed × group) for oxyHb concentration. Figure 3 shows the integral values of oxyHb concentrations in the ROIs where there was an interaction between task and group (task × group) under SSWS and FWS conditions. In the young-old, oxyHb significantly increased in the right PM, left S1, and bilateral PPC at both self-selected walking speed and fast walking speed (except for left PPC) during the DT conditions compared with ST conditions (*p* < 0.05). However, in the old-old, there was no significant change in the right PM, left S1, or bilateral PPC during DT conditions compared with ST conditions. In addition, the old-old presented lower oxyHb concentration than the young-old on bilateral PPC during DT conditions in SSWS (*p* < 0.05).

### 3.4. Association between Age and Regional oxyHb Value during Dual-Task Walking

**Figure 4:**
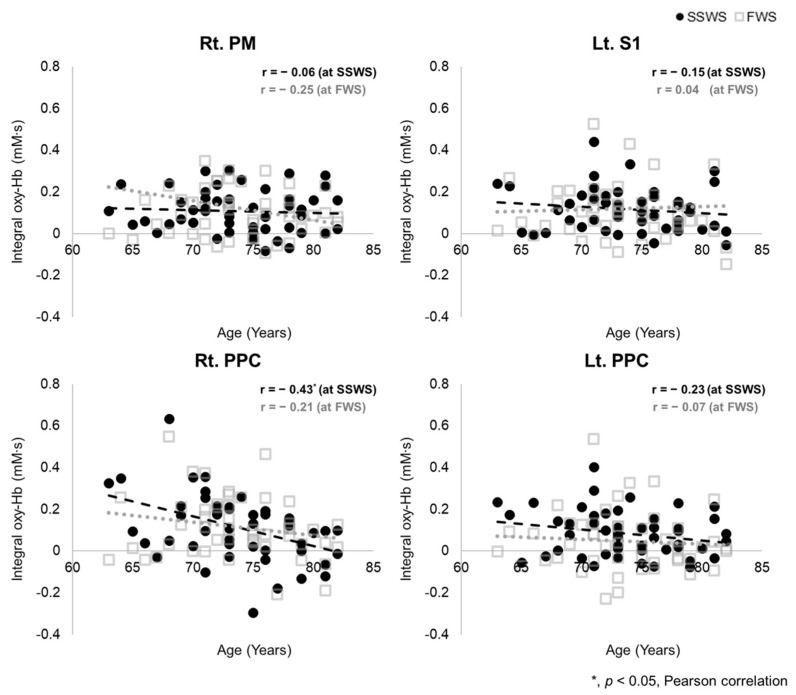
Association between age and regional oxyHb values during dual-task walking. The black circle represents self-selected walking speed (SSWS), and the gray square represents fast walking speed (FWS). In the SSWS, the integral value of oxyHb in the right PPC was negatively associated with age. There was no significant correlation in other ROIs such as the right PM, left S1, or left PPC. oxyHb, oxyhemoglobin; PM, pre-motor area; S1, primary somatosensory motor cortex; PPC, posterior parietal cortex; Rt., right; Lt., left.

Figure 4 shows the association between age and oxyHb concentration in ROIs that show interaction with task and group (task × group) during DT conditions. In the SSWS condition, the integral value of oxyHb on the right PPC was negatively associated with age (r = −0.43, *p* < 0.05). There was no significant correlation in other ROIs. In the FWS condition, there was no correlation between age and oxyHb value during DT conditions.

## 4. Discussion

In this study, we investigated the differences in cortical activity during walking between the young-old (65–74 years) and old-old (75–84 years) elderly. We first examined whether there were different functional profiles between the young-old and old-old using functional assessment. We then measured the effects of a simultaneous task and increased walking speed on cortical activation in the two groups. There were significant differences in functions such as walking ability and balance between the young-old and the old-old. The verbal fluency performance during dual-task walking was also significantly lower in the old-old than in the young-old at both self-selected and fast-walking speeds. Cortical activities were increased during dual-task conditions compared with single-task conditions but decreased during the fast-walking speed compared with the self-selected walking speed on the M1-leg, SMA, and dlPFC in both the young-old and the old-old. These oxyHb concentration changes were significantly less prominent in the old-old than in the young-old.

There were significant differences in gait and balance abilities between the young-old and old-old but no differences in sex ratio, height, and weight. The old-old group was slower in 10MWT and TUG than the young-old, and scores on the SPPB and FSST were lower than the young-old. Mobility inevitably decreases with age, and the decline tends to accelerate with age [20]. Previous gerontological studies have also reported significant differences in gait function in the young-old and the old-old groups based on the age of 75 years [44]. Pothier et al. reported an age-related decrease in dual-task cost and gait performance, and the performance of the old-old was more impaired than that of the young-old under high attention load conditions (i.e., walking under dual-task conditions) [25]. Therefore, it is valid to investigate changes in cortical activation by dual-task and gait speed conditions for two age groups with different profiles.

The cortical activity measured by oxyHb concentration in the motor and prefrontal area was increased during the dual-task walking compared with the single-task but decreased at fast walking speed compared with self-selected walking speed in both the young-old and the old-old elderly. Mixed ANOVA revealed a significant main effect of task on oxyHb concentration changes in the right M1-leg, bilateral PM, SMA, right PPC, bilateral dlPFC, and bilateral vmPFC. These results showed that the additional dual-task during walking increased the cortical activity of the prefrontal area, including the dlPFC and vmPFC, as well as the motor area including the M1-leg, PM, and SMA. The increases in prefrontal and motor activity in dual-task walking in the elderly have been described in previous studies [17,40,45]. These results corroborate the results of previous studies that supraspinal structures participate more actively in an environment that requires adaptive and attention control of movement [46,47]. Therefore, it seems like that additional activity was required in the cortical regions during dual-task walking in both age groups.

Also, the effect of walking speed was found in the left PPC and left vmPFC. When walking speed increased, cortical activity showed a tendency to decrease. Several studies have reported an increase in the PFC when walking speed increases, but these studies compared a slow walking speed (1.5 km/h) with a normal walking speed (3.0 km/h) [48]. Another study comparing self-selected walking speed and a speed 120 to 130% faster than SSWS reported a decrease in the PFC and M1 when walking at a fast speed in the elderly [41]. These results imply an increase in activity in areas governing automatic movement, such as subcortical and spinal structures, compared to supraspinal structures during high-speed walking [49].

The interaction effect between task and group (task × group) was shown in the right PM, left S1, and bilateral PPC. Especially, oxyHb concentration during dual-task walking at the self-selected walking speed in the right PPC was negatively associated with age. As reported in many studies, the PPC has an important role in gait adaptation [50,51]. There was also evidence that the PPC is involved in challenging gaits such as dual-task walking [48,49]. An et al. reported that unpredictable perturbations increased cortical activity in the SMA and PPC compared to normal walking [52]. Pizzamiglio et al. reported that gait speed and left PPC alpha (8–12 Hz) band were correlated when walking during conversation, and that mid-lateral trunk acceleration was predicted by left PPC beta (15–25 Hz) band when walking while texting [53]. Structural or functional defects of the PPC have been reported in elderly patients with gait impairment and in patients with Parkinson’s disease [54,55]. In this study, the cortical activity of the bilateral PPC was lower during dual-task gait in the old-old elderly compared to the young-old. In particular, the activity of the right PPC during dual-task walking had a negative linear relationship with age. The verbal fluency task performance during the dual-task was also lower in the old-old than in the young-old. It can be considered that the low PPC activity of the old-old had to do with the low dual-task performance during walking. This is similar to a previous study that reported a significant decrease in the size of P3 after age 80 during cognitive tasks compared to young adults [56]. These results imply that the PPC is utilized as an additional resource of compensatory activity in a gait environment that requires more attention, such as a dual-task, but that such a mechanism is not preserved after age 75 when gait function markedly declines. These results implied that the role of the PPC is very important to compensate for deterioration of gait function by aging.

There are some limitations in our study. First, we could not measure the task performance and gait ability during single- and dual-task walking. In the future, by comparing task performance and gait ability under single- and dual-task conditions, dual-task costs will be investigated. Also, it is necessary to investigate how changes in cortical activity with aging are related to task performance and gait ability. Second, since this study was conducted on a treadmill, there are some limitations in interpreting the results. Several studies have reported a difference in gait performance on the treadmill and overground, and that cortical activity was different [57]. This study had to be performed on a treadmill due to the communication limitations of the fNIRS device, which measures a wide brain area including the frontal, motor, and parietal lobes. In future studies, it is necessary to confirm the age difference in cortical activity during walking in an actual walking environment. Lastly, the change in cerebral blood flow was measured by setting the maximum source detector separation (SDS) to 30 mm without short SDS channels. As such, the recorded fNIRS signal reflects both extra- and intracerebral changes. Correction using short SDS signals can reduce the influence of skin blood flow changes. However, it was not applied to this study because there was no short SDS signal measurement equipment. Removal of skin blood flow should be considered in future studies [58].

## 5. Conclusions

This study demonstrated that changes in cortical activity occur during aging. In the elderly, the cortical activity during walking was affected by simultaneous activities or walking speed. These changes in cortical activity due to aging were shown in the old-old elderly as a marked deterioration in walking abilities. The bilateral PPC, which is responsible for gait adaptation, was not activated more highly in the old-old with impaired gait function than in the young-old. These different cortical plasticity profiles between the two groups of elderly might explain the neural background for impaired compensation strategies and the increased risk of falls with aging, suggesting needs for gait rehabilitation strategies for the elderly.

## Figures and Tables

**Table 1:** Demographic characteristics and functional differences between the young-old and the old-old elderly.

	All Participants (*n* = 50)	Young-Old (65–74 Years) (*n* = 27)	Old-Old (75–84 Years) (*n* = 23)	*p* Value
Age (years, mean ± SD, range)	73.80 ± 4.79 (65-82)	70.19 ± 3.03 (65–74)	78.04 ± 2.33 (75–82)	<0.001 *
Sex (Male:Female)	19:31	10:17	9:14	0.555
Height (cm, mean ± SD, range)	158.64 ± 7.67 (141–179)	160.22 ± 7.64 (148–179)	156.77 ± 7.45 (141–170)	0.114
Weight (kg, mean ± SD, range)	61.16 ± 9.23 (42–86)	63.19 ± 10.10 (42–86)	58.78 ± 7.64 (43–75)	0.093
BMI (kg/m^2^, mean ± SD, range)	24.22 ± 2.99 (16.2–31.2)	24.54 ± 2.93 (19.2–31.2)	23.85 ± 3.10 (16.2–28.4)	0.419
Education (years, mean ± SD, range)	8.31 ± 5.07 (0–16)	9.42 ± 3.09 (6–16)	7.65 ± 5.93 (0–16)	0.278
Medical History (N, %)				
Neck pain	5 (10.0)	4 (14.8)	1 (4.3)	0.229
Low back pain	9 (18.0)	5 (18.5)	4 (17.4)	0.606
Rheumatoid arthritis	2 (4.0)	1 (3.7)	1 (4.3)	0.713
Osteoarthritis	8 (16.0)	3 (11.1)	5 (21.7)	0.263
High blood pressure	28 (56.0)	14 (51.9)	14 (60.9)	0.362
Diabetes	9 (18.0)	5 (18.5)	4 (17.4)	0.606
Heart disease	5 (10.0)	1 (3.7)	4 (17.4)	0.129
SPPB (mean ± SD, range)	11.18 ± 0.98 (8–12)	11.59 ± 0.69 (10–12)	10.70 ± 1.06 (8–12)	<0.001 *
FSST (sec, mean ± SD, range)	8.10 ± 1.25 (4.48–10.94)	7.52 ± 1.12 (4.48–9.57)	8.78 ± 1.04 (6.85–10.94)	<0.001 *
TUG (sec, mean ± SD, range)	8.11 ± 1.20 (5.92–10.97)	7.66 ± 0.92 (5.92–9.50)	8.63 ± 1.28 (6.48–10.97)	0.003 *
10MWT (m/s, mean ± SD, range)	1.41 ± 0.20 (0.85–1.80)	1.48 ± 0.17 (1.22–1.80)	1.33 ± 0.21 (0.85–1.74)	0.006 *
K-MMSE (mean ± SD, range)	25.74 ± 3.50 (13–30)	26.63 ± 2.44 (22–30)	24.70 ± 4.26 (13–30)	0.063
K-MBI (mean ± SD, range)	100.0 ± 0.0 (100)	100.0 ± 0.0 (100)	100.0 ± 0.0 (100)	1.000
EQ-5D (mean ± SD, range)	0.89 ± 0.09 (0.56–0.95)	0.91 ± 0.08 (0.56–0.95)	0.86 ± 0.09 (0.67–0.95)	0.029 *
GDS-SF (mean ± SD, range)	3.58 ± 3.76 (0–13)	2.3 ± 3.34 (0–12)	5.09 ± 3.75 (0–13)	0.008 *

SD, standard deviation; BMI, body mass index; SPPB, short physical performance battery; FSST, four square step test; TUG, timed up and go test; 10MWT, 10-m walking test; K-MMSE, Korean Mini-Mental State Exam; K-MBI, Korean-modified Barthel index; EQ-5D, EuroQol-5 dimension; GSD-SF, geriatric depression scale short form; * and bold fonts, significant difference between the young-old and the old-old elderly (*p* < 0.05).

**Table 2:** Treadmill speed and verbal fluency performance during dual-task walking in the young-old and old-old.

	All Participants (*n* = 50)	Young-Old (65–74 Years) (*n* = 27)	Old-Old (75–84 Years) (*n* = 23)	*p* Value
Treadmill speed (km/h, mean ± SD, range)				
Self-selected walking	3.31 ± 0.45 (2.4–4.2)	3.41 ± 0.44 (2.4–4.2)	3.19 ± 0.44 (2.4–4.2)	0.035 *
Fast walking	4.31 ± 0.59 (3.1–5.5)	4.46 ± 0.57 (3.1–5.5)	4.14 ± 0.59 (3.1–5.5)	0.032 *
Verbal fluency performance (mean ± SD, range)				
Self–selected walking	29.79 ± 12.22 (9–61)	34.85 ± 15.24 (23–61)	26.83 ± 9.58 (9–45)	0.019 *
Fast walking	27.37 ± 9.95 (15–50)	33.43 ± 10.36 (25–50)	23.83 ± 8.14 (15–39)	0.037 *

SD, standard deviation; * and bold fonts, significant difference between the young-old and old-old elderly (*p* < 0.05).

**Table 3:** Changes in the integral values of oxyhemoglobin by walking condition in each ROI.

		Self-Selected Walking Speed				Fast Walking Speed				Main Effect			
		Single-Task		Dual-Task		Single-Task		Dual-Task		Task	Task × Group	Speed	Speed × Group
ROI		Young-Old	Old-Old	Young-Old	Old-Old	Young-Old	Old-Old	Young-Old	Old-Old				
M1-leg	Rt.	0.08 (0.02)	0.11 (0.02)	0.12 (0.02)	0.13 (0.02)	0.07 (0.02)	0.12 (0.03)	0.11 (0.02)	0.12 (0.04)	0.033 *	0.106	0.882	0.762
	Lt.	0.09 (0.02)	0.09 (0.02)	0.10 (0.02)	0.09 (0.02)	0.08 (0.02)	0.11 (0.03)	0.09 (0.03)	0.11 (0.04)	0.413	0.566	0.712	0.193
PM	Rt.	0.05 (0.02)	0.08 (0.03)	0.13 (0.02)	0.09 (0.02)	0.06 (0.03)	0.08 (0.02)	0.16 (0.05)	0.08 (0.02)	0.003 *	0.004 *	0.578	0.454
	Lt.	0.10 (0.03)	0.08 (0.03)	0.28 (0.07)	0.20 (0.07)	0.09 (0.03)	0.06 (0.03)	0.19 (0.03)	0.14 (0.04)	<0.001 *	0.436	0.079	0.878
SMA		0.05 (0.02)	0.08 (0.03)	0.11 (0.02)	0.09 (0.03)	0.04 (0.03)	0.06 (0.02)	0.09 (0.02)	0.10 (0.03)	<0.001 *	0.111	0.448	0.663
S1	Rt.	0.08 (0.02)	0.09 (0.02)	0.18 (0.03)	0.05 (0.03)	0.04 (0.02)	0.03 (0.05)	0.15 (0.03)	0.07 (0.03)	0.136	0.192	0.490	0.635
	Lt.	0.10 (0.02)	0.10 (0.02)	0.14 (0.02)	0.09 (0.02)	0.08 (0.02)	0.12 (0.03)	0.13 (0.03)	0.11 (0.05)	0.121	0.020 *	0.761	0.905
PPC	Rt.	0.09 (0.02)	0.08 (0.02)	0.18 (0.03)	0.05 (0.03)	0.04 (0.02)	0.03 (0.04)	0.15 (0.03)	0.07 (0.03)	0.006 *	0.008 *	0.110	0.510
	Lt.	0.07 (0.02)	0.05 (0.02)	0.11 (0.03)	0.05 (0.02)	0.03 (0.03)	0.05 (0.02)	0.06 (0.03)	0.03 (0.02)	0.333	0.045 *	0.001 *	0.127
dlPFC	Rt.	0.04 (0.02)	0.05 (0.02)	0.09 (0.02)	0.08 (0.02)	0.03 (0.02)	0.05 (0.02)	0.09 (0.02)	0.07 (0.03)	<0.001 *	0.118	0.656	0.773
	Lt.	0.04 (0.02)	0.05 (0.02)	0.06 (0.03)	0.07 (0.02)	0.03 (0.02)	0.03 (0.02)	0.06 (0.03)	0.07 (0.02)	0.014 *	0.859	0.070	0.102
vmPFC	Rt.	0.10 (0.03)	0.10 (0.04)	0.17 (0.03)	0.19 (0.05)	0.09 (0.03)	0.07 (0.02)	0.16 (0.03)	0.30 (0.08)	0.003 *	0.244	0.524	0.198
	Lt.	0.11 (0.02)	0.10 (0.02)	0.17 (0.03)	0.17 (0.03)	0.07 (0.03)	0.07 (0.02)	0.17 (0.03)	0.16 (0.04)	<0.001 *	0.889	0.008 *	0.145

Values represent mean (standard error); ROI, region of interest; M1-leg, primary leg motor cortex; PM, premotor area; SMA, supplementary motor area; S1, primary somatosensory cortex; PPC, posterior parietal cortex; dlPFC, dorsolateral prefrontal cortex; vmPFC, ventromedial prefrontal cortex; * and bold fonts, significant main effect using mixed-design ANOVA (*p* < 0.05).

## Data Availability

The data that support the findings of this study are available from the corresponding author upon reasonable request.

## References

[1] Shimada H., Kim H., Yoshida H., Suzukawa M., Makizako H., Yoshida Y., Saito K., Suzuki T. (2010). Relationship between Age-Associated Changes of Gait and Falls and Life-Space in Elderly People. J. Phys. Ther. Sci..

[2] Sudarsky L. (1990). Gait Disorders in the Elderly. N. Engl. J. Med..

[3] Studenski S., Perera S., Patel K., Rosano C., Faulkner K., Inzitari M., Brach J., Chandler J., Cawthon P., Connor E.B. (2011). Gait Speed and Survival in Older Adults. JAMA.

[4] Verghese J., Ma A.L., Hall C., Katz M.J., Ambrose A.F., Lipton R.B. (2005). Epidemiology of Gait Disorders in Community-Residing Older Adults. J. Am. Geriatr. Soc..

[5] Harada T., Miyai I., Suzuki M., Kubota K. (2008). Gait capacity affects cortical activation patterns related to speed control in the elderly. Exp. Brain Res..

[6] Mihara M., Miyai I., Hatakenaka M., Kubota K., Sakoda S. (2008). Role of the prefrontal cortex in human balance control. NeuroImage.

[7] Wilson J., Allcock L., Mc Ardle R., Taylor J.-P., Rochester L. (2018). The neural correlates of discrete gait characteristics in ageing: A structured review. Neurosci. Biobehav. Rev..

[8] Hsu C.L., Best J.R., Voss M.W., Handy T.C., Beauchet O., Lim B.C., Liu-Ambrose T. (2018). Functional Neural Correlates of Slower Gait among Older Adults with Mild Cognitive Impairment. J. Gerontol. Ser. A Boil. Sci. Med Sci..

[9] Lo O.-Y., Halko M.A., Zhou J., Harrison R., Lipsitz L.A., Manor B. (2017). Gait Speed and Gait Variability Are Associated with Different Functional Brain Networks. Front. Aging Neurosci..

[10] Holtzer R., Mahoney J.R., Izzetoglu M., Wang C., England S., Verghese J. (2015). Online fronto-cortical control of simple and attention-demanding locomotion in humans. NeuroImage.

[11] Plichta M.M., Herrmann M.J., Baehne C.G., Ehlis A.C., Richter M.M., Pauli P., Fallgatter A.J. (2006). Event-related functional near-infrared spectroscopy (fNIRS): Are the measurements reliable?. Neuroimage.

[12] Gramigna V., Pellegrino G., Cerasa A., Cutini S., Vasta R., Olivadese G., Martino I., Quattrone A. (2017). Near-Infrared Spectroscopy in Gait Disorders: Is It Time to Begin?. Neurorehabil. Neural Repair.

[13] Montero-Odasso M., Verghese J., Beauchet O., Hausdorff J.M. (2012). Gait and Cognition: A Complementary Approach to Understanding Brain Function and the Risk of Falling. J. Am. Geriatr. Soc..

[14] Al-Yahya E., Dawes H., Smith L., Dennis A., Howells K., Cockburn J. (2010). Cognitive motor interference while walking: A systematic review and meta-analysis. Neurosci. Biobehav. Rev..

[15] Bock O. (2008). Dual-task costs while walking increase in old age for some, but not for other tasks: An experimental study of healthy young and elderly persons. J. Neuroeng. Rehabil..

[16] Yogev-Seligmann G., Rotem-Galili Y., Mirelman A., Dickstein R., Giladi N., Hausdorff J.M. (2010). How Does Explicit Prioritization Alter Walking During Dual-Task Performance? Effects of Age and Sex on Gait Speed and Variability. Phys. Ther..

[17] Hamacher D., Herold F., Wiegel P., Hamacher D., Schega L. (2015). Brain activity during walking: A systematic review. Neurosci. Biobehav. Rev..

[18] Ohsugi H., Ohgi S., Shigemori K., Schneider E.B. (2013). Differences in dual-task performance and prefrontal cortex activation between younger and older adults. BMC Neurosci..

[19] Fraser S.A., Dupuy O., Pouliot P., Lesage F., Bherer L. (2016). Comparable Cerebral Oxygenation Patterns in Younger and Older Adults during Dual-Task Walking with Increasing Load. Front. Aging Neurosci..

[20] Forrest K.Y.Z., Zmuda J.M., Cauley J.A. (2006). Correlates of Decline in Lower Extremity Performance in Older Women: A 10-Year Follow-Up Study. J. Gerontol. Ser. A Boil. Sci. Med Sci..

[21] Shumway-Cook A., Guralnik J.M., Ms C.L.P., Coppin A.K., Ciol M., Bandinelli S., Ferrucci L. (2007). Age-Associated Declines in Complex Walking Task Performance: The Walking InCHIANTI Toolkit. J. Am. Geriatr. Soc..

[22] Thaler-Kall K., Peters A., Thorand B., Grill E., Autenrieth C.S., Horsch A., Meisinger C. (2015). Description of spatio-temporal gait parameters in elderly people and their association with history of falls: Results of the population-based cross-sectional KORA-Age study. BMC Geriatr..

[23] Magaziner J. (1989). Demographic and epidemiologic considerations for developing preventive strategies in the elderly. Md. Med. J..

[24] Gimmon Y., Rashad H., Kurz I., Plotnik M., Riemer R., Debi R., Shapiro A., Melzer I. (2018). Gait Coordination Deteriorates in Independent Old-Old Adults. J. Aging Phys. Act..

[25] Pothier K., Benguigui N., Kulpa R., Chavoix C. (2014). Multiple Object Tracking While Walking: Similarities and Differences between Young, Young-Old, and Old-Old Adults. J. Gerontol. Ser. B.

[26] Na H.R., Lee J.-W., Lee S.-H., Yang D.W., Han I.-W., Kim D.H., Yu K.-H., Lee J.S., Kim J.-S., Kim S. (2006). P3–076: The validity and reliability of the Korean version severe impairment battery. Alzheimer’s Dement..

[27] Guralnik J.M., Simonsick E.M., Ferrucci L., Glynn R.J., Berkman L.F., Blazer D.G., Scherr P.A., Wallace R.B. (1994). A Short Physical Performance Battery Assessing Lower Extremity Function: Association with Self-Reported Disability and Prediction of Mortality and Nursing Home Admission. J. Gerontol..

[28] Dite W., Temple V. (2002). A clinical test of stepping and change of direction to identify multiple falling older adults. Arch. Phys. Med. Rehabil..

[29] Podsiadlo D., Richardson D. (1991). The timed “Up & Go”: A test of basic functional mobility for frail elderly persons. J. Am. Geriatr. Soc..

[30] Collen F.M., Wade D.T., Bradshaw C.M. (1990). Mobility after stroke: Reliability of measures of impairment and disability. Int. Disabil. Stud..

[31] Lee D.Y., Lee K.U., Lee J.H., Kim K.W., Jhoo J.H., Youn J.C., Kim S.Y., Woo S.I., Woo J.I. (2002). A Normative Study of the Mini-Mental State Examination in the Korean Elderly. J. Korean Neuropsychiatr. Assoc..

[32] Alden D., Austin C., Sturgeon R. (1989). A Correlation between the Geriatric Depression Scale Long and Short Forms. J. Gerontol..

[33] Balestroni G., Bertolotti G. (2012). EuroQol-5D (EQ-5D): An instrument for measuring quality of life. Monaldi Arch. Chest Dis..

[34] Jung H.Y., Park B.K., Shin H.S., Kang Y.K., Pyun S.B., Paik N.J., Kim S.H., Kim T.H., Han T.R. (2007). Development of the Korean Version of Modified Barthel Index (K-MBI): Multi-center Study for Subjects with Stroke. J. Korean Acad. Rehab. Med..

[35] Wass E., Taylor N.F., Matsas A. (2005). Familiarisation to treadmill walking in unimpaired older people. Gait Posture.

[36] Fukuchi C.A., Fukuchi R.K., Duarte M. (2018). A public dataset of overground and treadmill walking kinematics and kinetics in healthy individuals. PeerJ.

[37] Dorfman M., Herman T., Brozgol M., Shema S., Weiss A., Hausdorff J.M., Mirelman A. (2014). Dual-Task Training on a Treadmill to Improve Gait and Cognitive Function in Elderly Idiopathic Fallers. J. Neurol. Phys. Ther..

[38] Tak S., Ye J.C. (2013). Statistical analysis of fNIRS data: A comprehensive review. NeuroImage.

[39] Benjamini Y., Hochberg Y. (1995). Controlling the False Discovery Rate: A Practical and Powerful Approach to Multiple Testing. J. R. Stat. Soc. Ser. B Methodol..

[40] Robbins D., Elwell C., Jimenez A., Goss-Sampson M. (2012). Localised Muscle Tissue Oxygenation during Dynamic Exercise with Whole Body Vibration. J. Sports Sci. Med..

[41] Herold F., Wiegel P., Scholkmann F., Müller N.G. (2018). Applications of Functional Near-Infrared Spectroscopy (fNIRS) Neuroimaging in Exercise–Cognition Science: A Systematic, Methodology-Focused Review. J. Clin. Med..

[42] Strangman G., Culver J.P., Thompson J.H., Boas D.A. (2002). A quantitative comparison of simultaneous BOLD fMRI and NIRS recordings during functional brain activation. Neuroimage.

[43] Morais G.A.Z., Balardin J.B., Sato J.R. (2018). fNIRS Optodes’ Location Decider (fOLD): A toolbox for probe arrangement guided by brain regions-of-interest. Sci. Rep..

[44] Ferraro K. (1980). Self-Ratings of Health among the Old and the Old-Old. J. Heal. Soc. Behav..

[45] Al-Yahya E., Mahmoud W., Meester D., Esser P., Dawes H. (2019). Neural Substrates of Cognitive Motor Interference during Walking; Peripheral and Central Mechanisms. Front. Hum. Neurosci..

[46] Abbud G., Li K., DeMont R. (2009). Attentional requirements of walking according to the gait phase and onset of auditory stimuli. Gait Posture.

[47] Meester D., Al-Yahya E., Dawes H., Martin-Fagg P., Piñon C. (2014). Associations between prefrontal cortex activation and H-reflex modulation during dual task gait. Front. Hum. Neurosci..

[48] Kim H.Y., Kim E.J., You J.S.H. (2017). Adaptive locomotor network activation during randomized walking speeds using functional near-infrared spectroscopy. Technol. Heal. Care.

[49] Nordin A.D., Hairston W.D., Ferris D.P. (2019). Faster Gait Speeds Reduce Alpha and Beta EEG Spectral Power From Human Sensorimotor Cortex. IEEE Trans. Biomed. Eng..

[50] Marigold D.S., Andujar J.E., Lajoie K., Drew T. (2011). Chapter 6—Motor planning of locomotor adaptations on the basis of vision: The role of the posterior parietal cortex. Prog. Brain Res..

[51] Young D.R., Parikh P.J., Layne C. (2020). The Posterior Parietal Cortex is Involved in Gait Adaptation: A Bilateral Transcranial Direct Current Stimulation Study. Front. Hum. Neurosci..

[52] An J., Yoo D., Lee B.-C. Electrocortical activity changes in response to unpredictable trip perturbations induced by a split-belt treadmill. Proceedings of the 2019 41st Annual International Conference of the IEEE Engineering in Medicine and Biology Society (EMBC).

[53] Pizzamiglio S., Abdalla H., Naeem U., Turner D.L. (2018). Neural predictors of gait stability when walking freely in the real-world. J. Neuroeng. Rehabil..

[54] Rosano C., Aizenstein H., Brach J., Longenberger A., Studenski S., Newman A.B. (2008). Special Article: Gait Measures Indicate Underlying Focal Gray Matter Atrophy in the Brain of Older Adults. J. Gerontol. Ser. A Boil. Sci. Med Sci..

[55] Rubino A., Assogna F., Piras F., Di Battista M.E., Imperiale F., Chiapponi C., Spalletta G., Meco G. (2014). Does a volume reduction of the parietal lobe contribute to freezing of gait in Parkinson’s disease?. Parkinsonism Relat. Disord..

[56] Daffner K.R., Sun X., Tarbi E.C., Rentz D.M., Holcomb P.J., Riis J.L. (2011). Does compensatory neural activity survive old-old age?. NeuroImage.

[57] Roeder L., Boonstra T.W., Smith S.S., Kerr G.K. (2018). Dynamics of corticospinal motor control during overground and treadmill walking in humans. J. Neurophysiol..

[58] Scholkmann F., Gerber U., Wolf M., Wolf U. (2013). End-tidal CO_2_: An important parameter for a correct interpretation in functional brain studies using speech tasks. NeuroImage.

